# Impact of an integrated nutrition, health, water sanitation and hygiene, psychosocial care and support intervention package delivered during the pre- and peri-conception period and/or during pregnancy and early childhood on linear growth of infants in the first two years of life, birth outcomes and nutritional status of mothers: study protocol of a factorial, individually randomized controlled trial in India

**DOI:** 10.1186/s13063-020-4059-z

**Published:** 2020-01-31

**Authors:** Sunita Taneja, Ranadip Chowdhury, Neeta Dhabhai, Sarmila Mazumder, Ravi Prakash Upadhyay, Sitanshi Sharma, Rupali Dewan, Pratima Mittal, Harish Chellani, Rajiv Bahl, Maharaj Kishan Bhan, Nita Bhandari, on behalf of the Women and Infants Integrated Growth Study (WINGS) Group, Farhana Rafiqui, Jasmine Kaur, Gunjan Aggarwal, Nikita Arya, Anita Kate, Medha Shekhar, Shruti Verma, Ratan Shekhawat, Neelam Kaur, Richa Chauhan, Kaavya Singh, Geeta Mehto, Runa Ghosh, Neha Tyagi, Payal Chakraborty, Ramanjit Kaur, Kunal Kishore, Navneet Mehra, Anuradha Tamaria, Ritu Chaudhary, Aparna Singh, Priyanka Singh, Dolly Jain, Gulafshan Ansari, Tivendra Kumar, Savita Sapra, Afifa Khatun, Kiran Bhatia, Manisha Gupta, Girish Chand Pant, Tarun Shankar Choudhary, Ankita Dutta, Deepak More, K. C. Aggarwal, Sujata Das, Pradeep Debata, Anita Yadav, Reeta Bansiwal, Abhinav Jain, Nitika

**Affiliations:** 1 Centre for Health Research and Development, Society for Applied Studies, 45, Kalu Sarai, New Delhi, India; 2 Vardhman Mahavir Medical College and Safdarjung Hospital, New Delhi, India; 3 Department of Maternal, Newborn, Child and Adolescent Health, World Health Organization, Geneva, Switzerland; 4 Knowledge Integration and Translational Platform (KnIT), Biotechnology Industry Research Assistance Council (BIRAC), Department of Biotechnology, Government of India, New Delhi, India; 5 Indian Institute of Technology, New Delhi, India

**Keywords:** Pre- and peri-conception, Pre-pregnancy, Stunting, Preterm, Small-for-gestation age, Low birth weight, Integrated intervention, Pregnancy interventions, Nutrition interventions, Growth, Intergenerational effect

## Abstract

**Background:**

The period from conception to two years of life denotes a critical window of opportunity for promoting optimal growth and development of children. Poor nutrition and health in women of reproductive age and during pregnancy can negatively impact birth outcomes and subsequent infant survival, health and growth. Studies to improve birth outcomes and to achieve optimal growth and development in young children have usually tested the effect of standalone interventions in pregnancy and/or the postnatal period. It is not clearly known whether evidence-based interventions in the different domains such as health, nutrition, water sanitation and hygiene (WASH) and psychosocial care, when delivered together have a synergistic effect. Further, the effect of delivery of an intervention package in the pre and peri-conception period is not fully understood. This study was conceived with an aim to understand the impact of an integrated intervention package, delivered across the pre and peri-conception period, through pregnancy and till 24 months of child age on birth outcomes, growth and development in children.

**Methods:**

An individually randomized controlled trial with factorial design is being conducted in urban and peri-urban low- to mid-socioeconomic neighbourhoods in South Delhi, India. 13,500 married women aged 18 to 30 years will be enrolled and randomized to receive either the pre and peri-conception intervention package or routine care (first randomization). Interventions will be delivered until women are confirmed to be pregnant or complete 18 months of follow up. Once pregnancy is confirmed, women are randomized again (second randomization) to receive either the intervention package for pregnancy and postnatal period or to routine care. Newborns will be followed up till 24 months of age. The interventions are delivered through different study teams. Outcome data are collected by an independent outcome ascertainment team.

**Discussion:**

This study will demonstrate the improvement that can be achieved when key factors known to limit child growth and development are addressed together, throughout the continuum from pre and peri-conception until early childhood. The findings will increase our scientific understanding and provide guidance to nutrition programs in low- and middle-income settings.

**Trial registration:**

Clinical Trial Registry – India #CTRI/2017/06/008908; Registered 23 June 2017, http://ctri.nic.in/Clinicaltrials/pmaindet2.php?trialid=19339&EncHid=&userName=society%20for%20applied%20studies

## Background

The major focus of the Millennium Development Goals was on reducing childhood mortality whereas the subsequent Sustainable Development Goals (SDGs) lay emphasis not only on improving survival but also on promotion of overall health and wellbeing of children [1, 2]. Adequate growth and development of the child lays the foundation of adult health and productivity and is an integral step towards achieving the SDGs. Evidence indicates that the first 1000 days of life i.e. from conception to two years of age are critical for optimal growth and brain development [3, 4]. Linear growth and neurodevelopment are particularly interlinked in the first two years of life as the etiology of poor growth (stunting) and neurodevelopment, such as insufficient nutrition, repeated infections and sub-optimal care, are similar during this period [5–7]. Birth weight, gestational age and size at birth are key parameters influencing growth and development in early life [8–10]. Low birth weight (LBW) resulting from both preterm birth and intrauterine growth retardation is a predictor of linear growth in early childhood and an important risk factor for stunting and poor cognitive development, in addition to its substantial contribution to mortality [11–13].

Most studies have tested the effect of standalone interventions within the first 1000 days window; primarily in the domains of health, nutrition, WASH and psychosocial health. These interventions were found to have modest effects on linear growth. The largest impact of a single intervention during pregnancy on birth weights was ~ 50 g mean difference (0.1 standard deviation; SD) and 15% reduction in those born LBW [14, 15]. The impact of a single intervention during pregnancy and/or the postnatal period on attained length was ~ 0.4 cm mean difference (0.1 SD) and ~ 15% reduction in stunting at 24 months of age was seen [16].

Valuable insights for the selection of study interventions were provided by an extensive literature review on interventions that influence birth, growth and development outcomes in children. Firstly, the causes are multifactorial and interventions are needed in the domains of health, nutrition, WASH and psychosocial care and support. Secondly, studies that tested the effect of standalone interventions found modest effects. It is yet uncertain whether the effects are synergistic, if interventions are delivered together as a package. Thirdly, interventions need to be delivered across the critical periods i.e. the 1000-days window from conception to birth and up to 24 months of child age. The review also revealed that the impact of delivering an intervention package covering the four different domains during the pre- and peri-conception period is yet unexplored [17].

Although the 1000 days period is indeed a critical window for the infant, mother’s own health, both physical and mental, as well as her nutrition at the time of conception is important for her own wellbeing and healthy birth outcomes and thriving of her child [18–20]. Poor nutrition in women of reproductive age and during pregnancy can impair fetal growth, which is associated with preterm birth and small-for-gestation (SGA) newborns [21, 22]. Undernutrition and deficiency of micronutrients such as iron, iodine and folic acid in women can have substantial effects on infant health and development outcomes [21, 23]. Hypothyroidism, hypertension and diabetes, and reproductive tract infections (RTI) also affect birth outcomes and subsequent infant survival, health and growth [24–27].

Observational data reveal a relationship between substance use in mothers, specifically of alcohol and tobacco, and maternal depression, with child growth and development [28–30]. The psychosocial health of women at the time of pregnancy may also influence fetal and infant growth [31].

Maternal height is an indicator of intergenerational linkages between maternal and child nutrition and health; short maternal height is associated with offspring undernutrition [32].

We are conducting an individually-randomized controlled trial in urban and peri-urban low- to mid-socioeconomic neighborhoods in Delhi, India to ascertain the impact that can be achieved on birth outcomes, growth and development in children by intervening before women become pregnant, in addition to delivering interventions during pregnancy and postnatal life. The study aims to achieve optimal growth and development in infants and children through concurrent delivery of an integrated package of evidence-based interventions covering the continuum from the pre- and peri-conception period to early childhood. We used a factorial design so that the impact of intervening during the pre-pregnancy period alone could also be assessed.

### Objectives

The primary objective is to determine the effect of integrated and concurrent delivery of interventions to improve health, nutrition, WASH and psychosocial status during the pre- and peri-conception period alone (pre- and peri-conception intervention package); during pregnancy and early childhood (enhanced antenatal, postnatal and early childhood care) and; throughout the pre- and peri-conception period, pregnancy and early childhood (pre- and peri-conception intervention package and enhanced antenatal, postnatal and early childhood care), on preterm birth, LBW and SGA, and stunting at 24 months of age compared to routine care and additionally, to assess whether the effect of these interventions differs by maternal stature (< 150 cm or ≥ 150 cm).

The secondary objectives are to determine the effect of the same package on nutritional status, morbidity and neurodevelopment in children, and on women’s nutritional status and morbidity in the pre- and peri-conception, pregnancy and postpartum periods.

## Methods

### Study design, setting and participants

The study is an individually randomized trial with a factorial design and is being conducted in urban and peri-urban low-to mid-socioeconomic neighborhoods of South Delhi, India [33]. In this setting, the proportion of infants born LBW (~ 25%), the stunting rates among under twos (~ 40%) and maternal undernutrition (body mass index [BMI] < 18.5 kg/m^2^) ~ 22%, are similar to the national average [34].

**Fig. 1:**
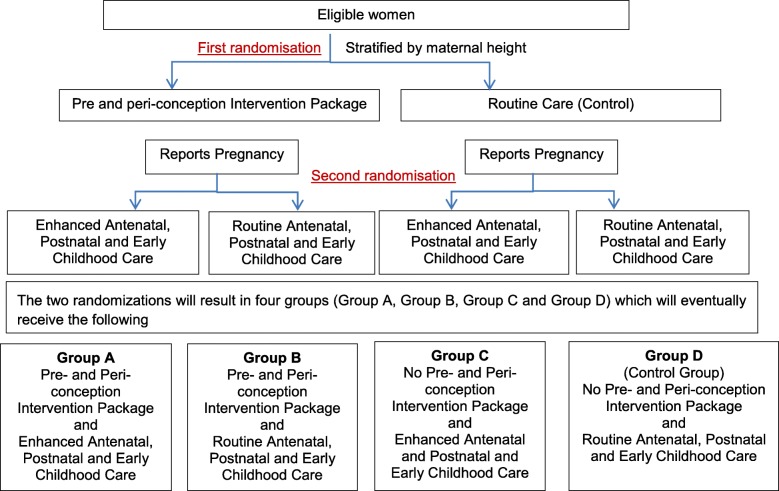
Study design

Eligible women are identified through a door-to-door survey. Those who consent for participation are enrolled (first randomization) and followed up until they are confirmed to be pregnant, or have completed 18 months of follow up post-enrolment. Once pregnancy is confirmed, consent is taken (second randomization) from the women for her and her infant’s participation in the trial (Fig. 1).

### Inclusion criteria

Women aged 18–30 years, married and living with their husband, with no or one child and wish to have a child, and consent for participation in the study.

### Exclusion criteria

Families who plan to move out of the study area or live in temporary housing (households without concrete roof, toilet, water connection and legal electricity) are excluded as they are likely to be relocated by the government in the near future.

### Sample size

Sample sizes were calculated for 90% power and 95% confidence level except for the preterm birth outcome for which the power is 80% for comparison between effect of pre- and peri-conception intervention package and enhanced antenatal, postnatal and early childhood care. Larger effect sizes than those shown for single interventions were assumed for the combined effect of pre- and peri-conception intervention package, enhanced antenatal, postnatal and early childhood care group [14–16]. A 1.5 times higher effect size for the impact of either pre- and peri-conception intervention package or enhanced antenatal, postnatal and early childhood care compared with control, and at least 2 times higher for combined effect of pre- and peri-conception intervention package and enhanced antenatal, postnatal and early childhood care compared to control was assumed (Tables 1 and 2).

**Table 1:** Sample size estimates for effect of pre- and peri-conception intervention package alone A + B vs C + D) or Enhanced antenatal, postnatal and early childhood care alone (A + C vs B + D)

	Main effect size	Sample size per two groups
Linear growth at 24 months		
- Mean length-for-age z-score (LAZ)	0.15 SD (absolute value 0.65 cm at 24 mo)	935
- Proportion stunted (30%)	25% relative reduction	772
Birth weight/birth length	0.15 SD (absolute value 75 g birth weight and 0.35 cm birth length)	935
Proportion LBW (25%)	25% relative reduction	918
Preterm birth (12%) [35]	25% relative reduction	2193
SGA at birth (36%) [10]	25% relative reduction	558

90% power and 95% confidence level for all outcomes

**Table 2:** Sample size estimates for the combined effect of pre- and peri-conception intervention package and enhanced antenatal, postnatal and early childhood care (A vs D)

	Main effect size	Sample size per group
Linear growth at 24 months		
- Mean LAZ	0.20 SD (absolute value 0.80 cm at 24 mo)	527
- Proportion stunted (30%)	30% relative reduction	491
Birth weight/birth length	0.20 SD (absolute value 100 g birth weight and 0.45 cm birth length)	527
Proportion LBW (25%)	30% relative reduction	624
Preterm birth (12%) [35]	30% relative reduction	1100
SGA at birth (36%) [10]	30% relative reduction	381

90% power for all outcomes except 80% for preterm births. 95% confidence level for all outcomes

We propose to enrol a total of 13,500 eligible women (6750 in pre- and peri-conception intervention package group and 6750 in control group) based on the following assumptions: 45% of reproductive-age women randomized get pregnant in the 18 months of the pre- and peri-conception intervention period, 30% loss (abortions, still births, maternal deaths, moving away, refusals) between pregnancy and live birth and 20% loss (loss-to-follow-up and child deaths) between birth and 24 months of age.

We will get ~ 1100 live births per group. This will enable us to detect a 25% difference in preterm births, proportion LBW and SGA between pre- and peri-conception package alone and control group (A + B vs C + D) and enhanced antenatal, postnatal and early childhood care alone and control group (A + C vs B + D) and a mean difference of 0.15 SD in LAZ score at 24 months for the above comparisons.

We will get at least 600 children in each of the four groups (A, B, C, D) at 24 months. We will be able to detect 0.2 SD difference in mean LAZ score at 24 months and 0.2 SD difference in birth weight and birth length between pre- and peri-conception intervention package and enhanced antenatal, postnatal and early childhood care and control group.

The sample size of ~ 2400 (at least 600 children in each of the 4 groups) at 24 months of age will also allow us to detect an interaction odds ratio (lOR) of ≥1.70 to 1.85 in the proportion of stunted children among short mothers (< 150 cm) and among tall mothers (≥ 150 cm) between the control (routine care) and the intervention group who received the intervention package throughout, with 80% power and 95% confidence level.

### Study interventions

The interventions are in four domains i.e. health, nutrition, psychosocial care and support, and WASH during pre- and peri-conception period, pregnancy and the postnatal (0 to 24 months) periods. These were selected based on evidence of their impact on preterm birth, SGA, birth weight and length and linear growth at 24 months, and finalized in consultation with the Technical Advisory Group (TAG) constituted for the study (Additional file 1). A summary of the intervention packages is shown in Table 3; details are given in Additional file 2.

**Table 3:** Intervention packages in the pre- and peri-conception period, pregnancy and postnatal period^a^

Period	Intervention group				Control group
	Health	Nutrition	Psychosocial care	WASH	
Pre- and peri-conception	Screen and treat medical conditions	Screen and treat malnutrition and anemia;	Promote positive thinking and problem-solving skills	Promote personal, menstrual and hand hygiene	Only weekly IFA supplementation as part of National Program (National Iron Plus Initiative)
		Provide iron-folic acid, multiple micronutrients, locally-prepared snacks, egg or milk			
Pregnancy	> 8 antenatal contacts, screen and treat medical conditions, calcium and vitamin D supplementation	Provide iron-folic acid, multiple micronutrients, locally-prepared snacks and milk, monitor weight	Promote positive thinking and problem-solving skills	Provide water filters, soap, hand washing station, disinfectant	Routine antenatal care
Postnatal	Empower family to identify danger signs and seek care early	0–6 mo: lactation support for early and exclusive breastfeeding	Promote early child play and responsive care	Provide play mat and potty	Routine Postnatal care
Childhood		6–24 mo: promote timely complementary feeding and continued breastfeeding, provide quality food, monitor inadequate weight gain			
Mothers (0 to 6 mo)	Facilitate postnatal visit at 6 weeks	Iron-folic acid, multiple micronutrients, calcium and Vitamin D, locally-prepared snacks and milk supplementation	Promote positive thinking and problem-solving skills	Provide water filters, soap, hand washing station, disinfectant	

^a^Electronic monitoring to track women and children with problems to improve intervention delivery across all periods

The principles underlying the selection of interventions for the pre- and peri-conception period are that women are infection-free, nutritionally-replete and in a positive state of mental health when they conceive.

Women are screened and treated for health conditions known to affect fetal and infant growth [17, 36, 37]. Around 50% women of reproductive age in the study setting are micronutrient deficient [38]. In consultation with the TAG, it was decided to give half to three-quarters of the recommended daily allowance (RDA) of the micronutrients daily which are known to contribute to optimal birth outcomes. Around 20% women of reproductive age group in this setting are undernourished [34]. These women are given food supplements in the form of a choice of snacks prepared locally: 500 Kcal and 6–10 g protein for women with BMI between 16 kg/m^2^ and 18.5 kg/m^2^ and double the amount for women with BMI < 16 kg/m^2.^ to be consumed daily. One egg or milk (180 ml) both containing 70 Kcal and 6 g protein is given to all women with BMI < 21 kg/m^2^ 6 days a week, as a source of high-quality protein.

For the care component, the intent is to identify the presence of stressors and manage them to the extent possible, so that women enter the pregnancy in a positive state of mental health. Further, with improved psychological wellness, women are more likely to adopt behaviours that are beneficial for them. The study intervention focuses on counselling women to “think healthy”. A counselling module has been developed through adaptation of the WHO Thinking Healthy Module [39]. The adapted version of the module emphasizes the basic five principles - empathetic listening, guided discovery using pictures, family engagement, problem solving and behavioural activation and is aligned to the local context which makes it easy to comprehend by the participant. All women in the intervention group are counselled to promote generic problem-solving skills, inculcate positive thinking and empower them in a way that they learn to devise strategies, within their prevailing circumstances, to overcome day-to-day stressors. Women are also screened for depressive symptoms, use of tobacco, exposure to second-hand smoke and alcohol use by the spouse and are managed and counselled accordingly. The WASH interventions during this period are limited to prevention of RTI through promotion of menstrual and personal, and hand hygiene.

During pregnancy, the intent is to screen and treat medical conditions known to affect fetal and infant growth [40–44]. As micronutrient deficiency is high in this setting; pregnant women are advised ~ 1 RDA of daily micronutrient supplementation daily throughout pregnancy [45–47]. To meet the additional energy and protein requirements, food supplements are given to all women with BMI < 25 kg/m^2^ [43]. The consensus by the TAG was to estimate additional requirements assuming a 12 kg weight gain during pregnancy [48] . The supplements are provided through a choice of locally-prepared snacks (containing cereal, pulses, soya, oil, sugar, salt, milk powder) - 210 kcal, 2 g protein in second trimester; 400 kcal, 21 g protein in third trimester [49, 50]. All women are also given milk (180 ml, 70 Kcal, 6 g protein) 6 days a week throughout pregnancy. Additionally, women with BMI < 18.5 kg/m^2^ are given 500 Kcal, 20 g protein in the form of a hot-cooked meal as the first meal in the morning. Weight gain is monitored monthly; those with inadequate weight gain defined based on Institute of Medicine Guidelines get a hot cooked meal (500 Kcal, 20 g protein), 6 days a week [48, 51, 52].

Evidence suggests that maternal psychosocial health during pregnancy is related to pregnancy complications, fetal growth and birth outcomes. Further, depression and mood disorders can impair an individual’s ability to make rational decisions and access health services. An adaptation of the WHO Thinking Healthy Module is used for counselling during pregnancy [39]. The content covers three broad domains: personal health of the mother, her relationship with other family members and her relationship with the child. The components of care intervention essentially remain the same as for the preconception period; however, the frequency of visits is aligned to the antenatal care visits. The WASH interventions during pregnancy (and continued during postnatal period) include improvement of drinking water quality through provision of water filters and storage bottles; reducing fecal load in the environment by providing detergents for cleaning toilets (if not available at home), and promotion of handwashing to reduce fecal transmission by placing a handwashing station in households where these are not available, counselling on correct handwashing technique and timing and provision of soap for handwashing. We did not attempt to make infrastructural changes for sanitation as these are not feasible in an individually randomized trial. We also did not intervene to increase the quantity of water due to ethical and social reasons.

During the postnatal period (first 6 months), mothers are encouraged to visit the delivery facility according to the follow up schedule advised by them. Micronutrient supplementation is continued. Women are provided locally-prepared snacks (500 kcal, 15 g protein) and milk (180 ml, 70 Kcal, 6 g protein) 6 days a week to meet the additional requirements during lactation [49]. For the care component, the intent is to decrease the risk of postpartum depression which has been shown to negatively affect breastfeeding performance and long-term child growth and development. Counselling is done using a module developed through adaptation of the WHO Thinking Healthy Module [39].

From birth to 6 months of age, mothers are counselled on early initiation of breastfeeding and exclusive breastfeeding. Monthly growth monitoring is done for early identification of growth faltering. Those with inadequate weight gain (<15th centile as per WHO weight velocity/month) are referred to the outreach clinic for a physician examination for morbidity and for lactation counseling. Micronutrient supplementation (calcium, iron, phosphorus, vitamin D) for LBW and very LBW infants is done according to WHO guidelines [53].

Caregivers of children aged 6 to 24 months are counselled on timely introduction of complementary foods at 6 months, on the frequency of feeding and types of food to be fed and their amounts, recipes of energy and nutrient-dense meals made from locally-available, culturally-acceptable foods are shared. Additionally a daily cereal mix packet (6 to 12 months; 125 Kcal per day, 2.5 g protein 12 to 24 months; 250 Kcal per day, 5 g protein that includes 80% to 100% RDA of micronutrients) is provided. This covers 40% to 60% of the energy requirement between 6 to 24 months of age assuming the child is breastfed [54]. Monthly weighing is continued to detect growth faltering. Those with inadequate weight gain (<25th centile according to WHO weight velocity/month) are referred to the physicians in the outreach clinic for assessment of morbidity. Additional food supplements in the form of snacks providing (~ 125 Kcal per day, ~ 2.5 g protein during 6 to 12 months; ~ 250 Kcal per day, ~ 5 g protein during 12 to 24 months) are offered.

Age-specific child play and stimulation activities, soon after birth, along with early identification of developmental deviations and their prompt management are the core components of the child-care package as brain growth is highly dynamic in the first two years of life and structured stimulation provided soon after birth by the mother and family members would accelerate development. The interventions for early child development have been adapted using Care for Child Development manual developed by WHO and UNICEF [55]. The pregnancy WASH interventions are continued in the postnatal period. Additional interventions are promotion of safe disposal of child feces and providing a potty at ~ 1 year of age. To promote a clean area for children, a play mat is provided ~ 6 months of age.

An electronic monitoring system has been developed to track women and children with problems and those who require additional support to achieve high compliance to interventions delivered during all the periods.

All participants (women and children) in both the groups are free to access the usual care pathways including free services from the government health system.

### Outcome measures

The primary outcomes include proportion preterm birth (ultrasound-confirmed gestational age at birth < 37 completed weeks); proportion SGA (birth weight centile <10th as per INTERGROWTH-21 standard) on day 7 of birth; proportion LBW (birth weight < 2500 g); mean birth weight and length; attained length (LAZ) at 24 months of age and proportion stunted (LAZ < -2 SD).

The list of secondary outcomes along with the timing of measurements during the pre- and peri-conception, pregnancy and postnatal periods are provided in Additional file 3.

The key secondary outcomes for children are proportion stunted at 6 and 12 months, wasted and underweight at 6, 12 and 24 months, weight and length trajectories from birth to 24 months, body composition (in a subsample) at 1 month of age and neurodevelopment at 6, 12, 18 and 24 months (in a subsample), micronutrients and anemia status at 24 months, morbidity and hospitalization from birth to 24 months (Additional file 3).

The key secondary outcomes for women are micronutrients and anemia status, depressive symptoms and infection at the end of pre- and peri-conception, during pregnancy and postpartum period (Additional file 3).

### Study procedures

#### Screening and enrollment

The screening and enrolment team (SET) identify eligible women through a door-to-door survey in the study areas of Dakshinpuri, Govindpuri, Madangir and Tigri, Khanpur, Sangam Vihar, Jaitpur and Meethapur and Madanpur Khadar areas of South Delhi. Information about the study is shared with families and written informed consent is taken from those who meet the inclusion criteria and are willing to participate in the study. Height (Seca-213 stadiometer) and weight (Salter 9509 weighing scale) measurements are taken and the participant is allocated to the intervention (pre- and peri-conception) or control (routine care) group [56, 57]. The intervention delivery team is informed if the woman is randomized to the intervention group.

Post-enrolment, information is documented on sociodemographic characteristics and the enrolled woman is requested to inform the study team by calling designated phone numbers if she gets pregnant. Workers from the SET make calls to all women every month (or home visits if the call is unsuccessful) to enquire about missed periods. If women report two missed periods or inform that they are pregnant (self-testing using a pregnancy kit), a trans-abdominal ultrasound is scheduled.

### Randomization, allocation and masking

The randomization list was prepared by an independent statistician at World Health Organization (WHO) using random permuted blocks, stratified by maternal height [< 150 cm (< − 2 SD) and ≥ 150 cm (≥ − 2 SD)] of the WHO standards [58].

The first randomization is done at enrollment when married women aged 18 to 30 years fulfil the eligibility criteria and consent to participate in the study. The second randomization is done when women become pregnant during the 18 months follow up period, and are eligible (not moving away from the study area) at rescreening and consent to their own and their baby’s participation in the study. The group allocation is done through a web-based system. There are no additional criteria for discontinuation or modification of allocated interventions.

Masking participants and study teams is not possible because of the nature of interventions in this trial. However, attempts are made to keep the independent outcome ascertainment team unaware of the group allocation, to the extent possible.

### Intervention delivery

#### Pre- and peri-conception period

The intervention delivery team conducts the first visit post enrolment and three-monthly follow up visits thereafter for a period of 18 m or till the women gets pregnant. At the first visit, symptoms of RTI and tuberculosis (TB) are ascertained and a history of epilepsy is taken. Blood pressure is measured (Omron 1300 digital blood pressure device) [59]. A blood specimen taken to check for anemia (hemoglobin - Hb), diabetes (Glycated hemoglobin - HbA1c), thyroid disorder (Thyroid-stimulating hormones - TSH) and syphilis (Rapid plasma regain; RPR).

Women with severe anemia (Hb < 8 g/dl), prediabetes (HbA1c 5.7% to 6.4%), diabetes (HbA1c ≥6.5%), high blood pressure (≥140/90 mmHg; at least two measurements 48 h apart), hypo- (TSH > 5.5 IU/mL) or hyper-thyroidism (TSH < 0.4 IU/mL), presence of symptoms of suspected TB, RPR positivity, reporting of symptoms of RTI or sexually transmitted diseases (STI), epilepsy and severe undernutrition (BMI < 16 kg/m^2^) are referred to the collaborating tertiary care hospital (Safdarjung Hospital). An outreach clinic manned by study physicians, nutritionists, psychologists and laboratory staff has been set up in the study area. Women with mild to moderate anemia (Hb 8 to < 12 g/dL) are treated with iron-folic acid for three months or till they are non-anemic. Women with RTI or STI are managed in the outreach clinic or hospital using algorithms of the syndromic approach [60]. Family planning advice is offered to recently (< 1 year) married women, those with a young (< 1 year) child and if women have moderate to severe anemia, symptoms and signs of RTI/STI, undernutrition, hypo- or hyper-thyroidism and diabetes requiring treatment.

Women are screened for depressive symptoms using the Patients Health questionnaire (PHQ-9) and are managed according to the severity of depressive symptoms and whether they have suicidal ideation [61]. All women are counselled on positive thinking and problem-solving skills [39]. Women are also counselled against tobacco use (smoke and smokeless form) and on ways to reduce exposure to second-hand smoke. All women in the intervention group are counselled at home, using the module described earlier [39]. Those with moderate depressive symptoms (PHQ-9 score 10–14) are counselled in the outreach clinic by a trained psychologist. For women with a score of 15 or more or those reporting suicidal thoughts, urgent referral to a psychiatrist is facilitated.

Family members who smoke inside the house are counselled with the intention of motivating them to either decrease or quit smoking and if that is not possible, then avoid smoking inside the house or in the vicinity of the women. If a woman permits a male counsellor to meet the spouse for his drinking habits, a study team member approaches the spouse at a convenient time and administers the AUDIT tool to assess severity of alcohol use [62]. Referral is facilitated to a tertiary care hospital for those with a score of ≥20; in those with a score of < 20, counselling is done at home on ways to quit or reduce alcohol use.

Women are counselled on personal, menstrual and hand hygiene.

This team contacts each woman three-monthly for the following:

- Follow up investigations for illnesses diagnosed at the previous visit
- Ascertainment of symptoms of RTI
- Administration PHQ 9 to screen for depressive symptoms and counselling if needed
- Assessment of use of tobacco by woman exposure to second hand smoke and alcohol use in spouse and counselling, if needed
- Counselling on personal, menstrual and hand hygiene.

After 12 months of enrolment, a blood specimen is obtained from all women in the study except those who have become pregnant and the investigations done at baseline are repeated.

#### Follow up visits in the pre- and peri-conception period for the intervention group

Study community workers – the *Sangini (*“friend”) similar in background to government community workers - Accredited Social Health Activists (ASHA; http://www.nhm.gov.in/communitisation/asha/about-asha.html) visit enrolled women at least once a week throughout the follow up period of 18 months or till women become pregnant. They counsel on study interventions, observe intake of supplements when possible, replenish supplies and organize referrals to hospital and the outreach clinic, when necessary. “High risk” women i.e. those with moderate to severe anemia, hypo- and hyper-thyroidism, symptoms and signs of RTI/STI and undernutrition, are visited more often. These visits also provide women an opportunity to discuss their personal problems with the *Sangini*. Eggs or milk are delivered 6 days a week through neighborhood depots managed by women residing in the study communities. These women visit enrolled participants and attempt to observe the intake of egg or milk. If the woman is not available, repeat visits are made. The intake by each woman is documented every day.

#### Pregnancy, postnatal and early childhood period after the second randomization

When a pregnancy is reported, trans-abdominal ultrasound (GE ultrasound Voluson S8, PI Healthcare Inc., 23865 Via Del Rio, Yorba Linda CA 92887, USA) is done between 9 and 13 weeks of gestation. If women are eligible at rescreening (not moving away from the study area) and consent is given for further participation in the study, they are randomized to either receive Enhanced Antenatal, Postnatal and Early Childhood Care (national and/or WHO-recommended interventions delivered well - intervention group) or routine care (control group). Each woman is allocated to a worker in the intervention delivery team for pregnancy. This worker visits the women monthly and ensures registration in Safdarjung Hospital (if the woman does not want to go to Safdarjung Hospital, she is encouraged to register in a hospital of her choice) for delivery, counsels on the importance of regular antenatal care, danger signs during pregnancy, on the benefits of an adequate diet during pregnancy and preparation for breastfeeding and infant care and promotes institutional delivery [43]. The workers encourage the woman for timely antenatal care (1 visit in the first trimester, 2 in the second and 5 in the third trimester). They also ensure uninterrupted supplies of iron folic acid, calcium, vitamin D and multiple micronutrient supplements to the woman throughout pregnancy [43].

Milk is delivered to pregnant women through neighbourhood depots; an attempt is made to observe the intake and compliance is documented at each visit.

Women are encouraged to consume snacks given to them (details given earlier). Monthly weight measurements are taken at home or at the outreach clinic.

The collaborating hospital follows the WHO recommendations on antenatal care [43]. The first visit for registration and the visits in the last trimester are done in Safdarjung Hospital. Women with complications (hypertension, gestational diabetes, severe anemia, previous bad obstetric history etc) are encouraged to visit the hospital for all antenatal visits; those without complications may use the outreach clinic.

*Sanginis* counsel pregnant women on positive thinking and problem solving skills using the module described earlier [39]. The use of tobacco by woman, exposure to second-hand smoke and alcohol use in the spouse is ascertained. The ensuing counselling is similar to that in the pre-conception period. Women are screened for depressive symptoms using the PHQ-9 questionnaire four times during the pregnancy (once in the first trimester, twice in the second and once in the third trimester) [61]. The management strategy is similar to the preconception phase.

The team also counsels on the WASH interventions i.e. on personal and hand hygiene and use of safe drinking water from water filters.

Post-birth, the *Prerna (*“inspiration”) workers conduct follow up visits to the intervention group households. The first visit is made within 24 h of hospital discharge or birth in case of home delivery. Mothers are encouraged to comply with postnatal visits advised by the hospital for themselves, encouraged to consume milk, iron-folic acid, calcium, vitamin D and multiple micronutrients for the first 6 months post-delivery.

Home visits for all births are made by *Prerna* on days 3, 7, 10, 14, 28, and monthly from 2 to 24 months [63, 64]. Additional visits are made for those born preterm, LBW and for mothers with breastfeeding problems. Exclusive breastfeeding is promoted for the first 6 months. If the mothers report breastfeeding problems, visits by the study lactation counsellors are arranged. The *Prerna’s* counsel mothers on positive thinking and problem solving skills using the module described earlier. During these visits, the team also assesses the use of tobacco by woman, exposure to second-hand smoke and alcohol use in spouse. A brief questionnaire (Patient Health Questionnaire-2) is administered to women within a week of delivery to assess her mood and screen for depression [65].

The *Prerna* demonstrates to the families ways to interact and communicate with their babies. They also assess key developmental milestones at specified ages. Infants who do not attain age-specific milestones are referred to the paediatricians and psychologists.

Mothers are counselled to initiate complementary feeding at 6 months while continuing to breastfeed till 24 months of age. Recipes are shared on foods that can be prepared at home, along with ways to encourage the infant to eat. Packets of milk-cereal mix are provided to all children. Intake of daily iron supplementation is encouraged.

Monthly weight and length measurements are taken by the *Prerna*. Infants (0 to 6 months) with inadequate weight gain (<15th centile according to WHO weight velocity/month) are visited by a lactation counsellor and referred to the physician at the outreach clinic if morbidity is present. Children between 6 to 24 months of age with inadequate weight gain (<25th centile according to WHO weight velocity/month) are referred to outreach clinic for examination by physicians. Additional locally-procured snacks or extra packets of foods are provided. Children with severe acute malnutrition (weight-for-length Z-score, WLZ < -3 SD) are managed at facilities.

Six-monthly deworming is given to all children from 12 months of age. Families are counselled about timely immunization, taught to recognize danger signs and to seek care promptly when the infant is ill.

The *Prerna’s* counsel on WASH interventions as during pregnancy. Additionally, counselling is done on hand hygiene for child feeding, safe disposal of feces, use of diapers and their disposal, and use of clean play area.

#### Process evaluation

Observed and independent observations are done for each worker at least once a month. Different activities (delivery of interventions and counselling for consumption of nutrient supplements, coping strategies, personal hygiene, hand washing practices, timely antenatal visits, optimal breast feeding practices and immunization in childhood, etc) conducted by the workers are observed by accompanying them. During these visits, the following aspects are evaluated; the quality of interaction between the workers and the family, whether the questions in the forms are asked correctly, quality of counselling and whether all procedures are performed as planned. During independent visits, the participants are asked whether the worker visited, the activities performed at the visit and the counselling provided.

The study also has a “Program Learning Team” which comprises of two social scientists who conduct interviews and observations around the key study activities. They assess compliance to interventions through interviews and observations. Additionally, in-depth interviews are done with non-responders to anemia and those with inadequate weight gain to ascertain possible reasons. The findings are communicated to the relevant study teams and are used to strengthen intervention delivery through retraining of workers and improvement in processes, whatever is applicable.

#### Outcome ascertainment

Participants in both groups are visited by the independent outcome ascertainment team at the end of pre- and peri-conception period. The symptoms of RTI, depressive symptoms, compliance to interventions, micronutrient, anemia, thyroid and diabetes status are ascertained. During pregnancy, weight gain, compliance to interventions and micronutrient status is assessed. Weight, length, head- and mid-upper arm circumference, child care practices, prevalence of illness and care seeking and hospitalization are assessed during early childhood (Additional file 3).

Weights and lengths are taken by pair of workers using digital weighing scale (model 354; Seca, California, USA) and infantometer (model 417; Seca, California, USA) to the nearest 10 g and 0.1 cm, respectively. Head and mid-upper arm circumference is taken using a measuring tape (model 212; Seca, California, USA) [66–68].

#### Laboratory investigations

At enrolment, ~ 10 ml blood sample is obtained from women in the intervention group to screen for anemia, thyroid disorders, diabetes, RPR and micronutrient assays. Around 10 ml blood is taken from women in both intervention and control groups when they report pregnancy or do not get pregnant during 18 months of the pre- and peri-conception period, in the third trimester of pregnancy and at 6 months postpartum from women in the intervention and control groups for anemia assessment, micronutrient status and inflammatory markers. 5 ml blood will be collected from children at 24 months of age in both groups for micronutrient assays.

The samples are centrifuged and serum is stored in a − 80 °C deep freezer in the “Clinical and Research Laboratory” set up in the field office, which is accessible only to the laboratory managers. Laboratory investigations are done in an accredited commercial laboratory (Strand-Quest Diagnostics Laboratory) and at referral hospitals (Safdarjung Hospital). The body composition analysis will be done by isotope dilution technique using deuterium oxide (2H2O) at 1 month of age in a subsample [69]. The infant saliva samples will be analyzed for enrichment of deuterium by Fourier transform infrared (FTIR) spectrophotometry [69].

#### Ultrasounds for assessment of pre term birth and fetal growth restriction

Ultrasounds in enrolled women during pregnancy (intervention and control groups) are done at the designated ultrasound centers. A trans-abdominal ultrasound is scheduled between 9 and 13 weeks of gestation to estimate gestational age calculated by fetal crown-rump length (CRL). If CRL is > 95 mm, femur length and head circumference will be used to assess gestational age [70]. Repeat ultrasounds are done at 26–28 weeks and 35–36 weeks of gestation and biparietal diameter (BPD), occipito-frontal diameter (OFD), head circumference (HC, using ellipse facility), abdominal circumference (AC using ellipse facility) and femur length (FL) are measured. All measurements are taken by trained radiologists according to the INTERGROWTH-21 standards [71].

Three measurements are taken for each fetal biometric variable: CRL, BPD, OFD, FL, HC and AC, with the woman in the lateral recumbent position [71]. The radiologists performing the scan are blinded to the group allocation of the pregnant women. All digital images are stored in a secured server. Images from 10% of all study participants are randomly selected and sent for external review for quality assurance.

#### Training and standardization

Prior to study initiation, all staff were trained in the study objectives, study strategy and in good clinical practices [72]. Additionally, each team underwent intensive training in their area of work (door-to-door survey, consenting, anthropometry measurements, assessment of morbidity, nutritional and psychosocial counselling, etc).

Inter- and intra-observer standardization exercises for weight, length, height, mid upper arm circumference and head circumference were conducted before study initiation for the SET, the independent outcome ascertainment team and for all other workers who take weight and length measurements; these are repeated 6 monthly. Weighing scales and infantometers are calibrated daily using standard weights and length measurement rods.

#### Study oversight and monitoring

Coordinators designated for each activity oversee the work of their teams. Weekly status reports are shared with the investigators. Periodic reviews meetings are organized between the study teams, coordinators and the investigators.

The WHO, Geneva and Biotechnology Industry Research Assistance Council (BIRAC), Department of Biotechnology, Government of India are responsible for oversight of the study. Technical staff from WHO and Bill & Melinda Gates Foundation interact with the investigators through conference calls once in two months and twice-yearly site visits to review study progress.

#### Data management

Data are captured electronically on tablets and mobile phones. Range and logical checks are built in to ensure data quality. The data management centre is set up in the field office. Real time data are transferred to the server. Logical errors and checks across different forms are run twice weekly; queries generated are given to study team for resolution and corrections incorporated.

#### Data and safety monitoring committee

A Data Safety Monitoring Committee (DSMC) has been constituted by the WHO and BIRAC to review the data, monitor the progress of the trial and assess safety of the interventions. The committee reviews data twice a year. The members include - an epidemiologist, a statistician and a clinician and a social scientist. An interim analysis will be conducted in a blinded manner when 50% of the babies in the study are born, a second review will be done when 50% of the babies in the study reach 2 years of age. The committee will advise the team on study continuation, modification or termination based on pre-decided stopping rules. This is a low-risk trial and serious adverse events are not anticipated. However, all deaths in enrolled participants are being reported to the local ethics committee and the World Health Organization for further communication to the DSMC. Additionally, adverse events reported with any supplement are being documented and all severe adverse events will be reported to the ethics committees and the DSMC.

### Statistical analyses

#### Primary comparisons

The primary analysis will be factorial, for comparison of the pre- and peri-conception intervention package (pre- and peri-conception intervention package alone or with enhanced antenatal, postnatal and early childhood care package i.e. Group A + B) with no pre- and peri-conception intervention package (enhanced antenatal, postnatal and early childhood care package alone or routine care i.e. Group C + D), and the enhanced antenatal, postnatal and early childhood care (with pre- and peri-conception intervention package or enhanced antenatal, postnatal and early childhood care alone i.e. Group A + C) with no enhanced antenatal, postnatal and early childhood care (pre- and peri-conception intervention package alone or routine care i.e. Group B + D) on birth outcomes (mean birth weight and length, proportion stunted at birth, proportion of babies born preterm and spontaneous preterm births, SGA and LBW) and mean length for age z-score and proportion stunted at 24 months.

In addition to the factorial analysis, we will also examine the impact of the combined pre- and peri-conception intervention package and enhanced antenatal, postnatal and early childhood care (Group A) on birth outcomes and attained length at 24 months of age, compared to routine care (Group D).

We will examine if there is an interaction between the intervention package delivered during peri-preconception period and that delivered during antenatal and early childhood period on primary outcomes.

#### Definitions of primary outcomes

Birth weight will be defined as weight taken by the study team at day 7 after birth and birth length as length taken by the study team any time within the first 7 days after birth to ensure comparability across the groups. Weight taken on day 0 is available for large proportion of infants. However, this proportion is likely to be unequal between the intervention groups (A + C) compared to control groups (B + D) because of greater ability to contact the intervention group mothers immediately after birth. We will therefore use weight on day7 taken consistently on the same day for both the groups as a measure of birth weight.

LBW will be defined as weight < 2500 g on day 7 after birth; proportion stunted as length-for-age z-score < − 2SD according to WHO standards [58]. Gestation at birth will be estimated by subtracting date of birth from date of dating ultrasound and adding it to gestational age as assessed by dating ultrasound according to INTERGROWTH-21 [70]. Preterm births will be defined as births occurring at < 37 completed weeks of gestation. Spontaneous preterm births will be defined as births occurring at < 37 weeks of gestation and preterm pre-labor rupture of membranes or spontaneous onset of labor.

Birth weight centiles will be calculated using the INTERGROWTH-21 standard based on day-7 weight and gestational age at birth [70]. SGA will be defined as birth weight centile <10th and < 3rd as per INTERGROWTH-21 standard [70]. Length-for-age z-score will be calculated based on length measured at 24 months (±28 days) and proportion stunted will be defined as those with length-for-age z-score < − 2 SD by WHO standards [58].

#### Flow of participants

**Fig. 2:**
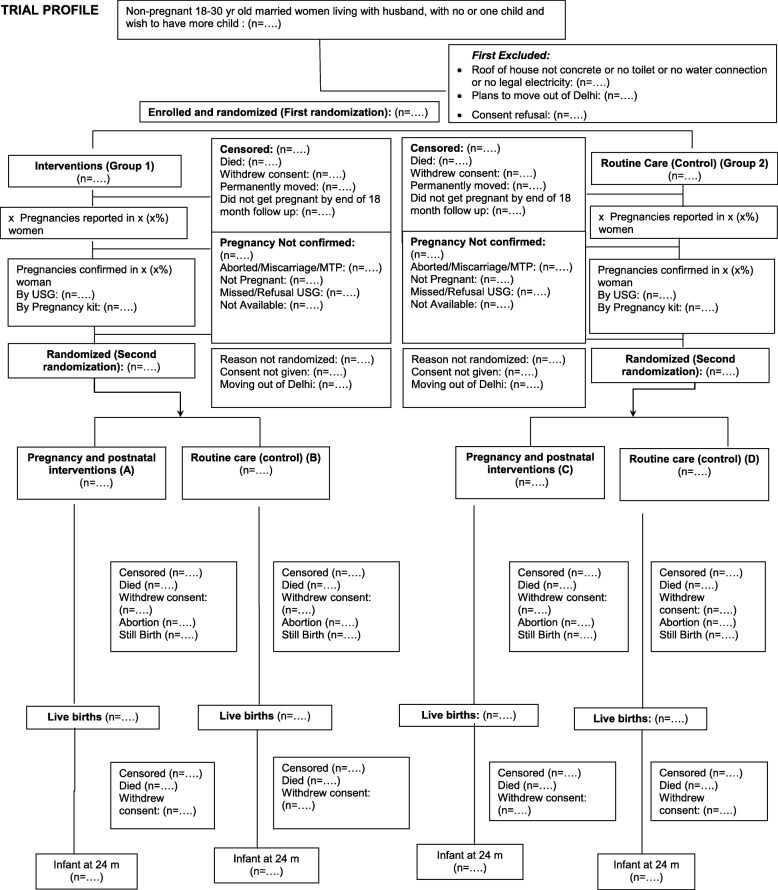
Trial profile

The flow and number of participants through assessment of eligibility, randomization, follow-up, and analysis will be presented (Fig. 2) along with reasons for exclusions and withdrawals for all time points.

#### Comparability between the two groups

Summary values (means, proportions) for sociodemographic characteristics among the groups will be presented in the baseline table. We will not perform any test of significance. Our large sample size is likely to yield a balance between the groups. However, we will carefully examine the size of any baseline differences. Imbalanced characteristics that may influence the primary outcomes will be adjusted appropriately.

#### Main effects

Analysis will be done by intention-to-treat. Mean (SD) birth weight, birth length, LAZ score at 24 months, proportion of preterm birth and spontaneous preterm birth, SGA, LBW, stunting at birth and 24 months will be presented for the different groups.

For binary outcomes, generalized linear models (GLMs) of the binomial family with a log-link function will be used to calculate the effect size [relative risk and 95% confidence interval (CI)]. For continuous outcomes, GLMs of the Gaussian family with an identity-link function will be used to calculate the effect size (difference in means and 95% CIs). The effect of interventions on secondary outcomes will be assessed using the same models as for primary outcomes.

#### Weight and length growth trajectories between birth and 24 months

A multivariable linear mixed-effect regression model with an unstructured covariance matrix will be used to examine the effect of the interventions on weight and length trajectories from birth to 24 months [73]. In this model, in order to account for the interdependence of multiple observation periods in the same child, time (age in months) of assessment will be taken to be the level-1 source of variation, with individual children at level 2. All potential covariates will be included as fixed effect variables in this model. The interaction between maternal stature and time (age in months) of assessment on different anthropometric outcomes i.e. LAZ, WAZ (weight-for-age z-score) and WLZ scores will be examined. If significant, the interaction term will be included in the model to obtain the independent effect of maternal stature at different ages.

#### Pre-specified subgroup analysis

We will conduct subgroup analysis for women according to their height (< 150 cm and ≥ 150 cm), underweight, BMI status at enrolment and at the time of pregnancy confirmation, years of education and high risk pregnancy. We will also conduct subgroup analysis by wealth quintile of the household. The relative measures of effect within each of these subgroups will be estimated.

The effect of interventions on secondary outcomes will be assessed using the same models as for primary outcomes.

## Discussion

This study is conceived with the underlying belief that investing efforts in promoting growth and development in children could potentially drive the transformation aimed to be achieved by 2030 under the SDG3 i.e. good health and wellbeing for all [1]. Existing evidence supports that the first 1000 days of life i.e. from conception to two years are critical for optimal growth and brain development but may not be sufficient [3, 4]. Studies have examined the effects of individual interventions on birth outcomes and early childhood growth and development, targeting the pregnancy period and/or the postnatal period and have observed low to modest effects sizes. Observational studies indicate that health prior to conception (pre- and peri-conception period) is linked to birth outcomes and could influence health across generations [17, 74]. However, intervention trials are yet to substantiate the initial observations and reliable evidence is required.

The study has been designed to examine the effect of an integrated package of evidence based interventions in four key domains namely health, nutrition, WASH and psychosocial care on birth outcomes, growth and development in early childhood. The health of the mother and her baby are strongly interlinked and therefore the interventions have been selected so that they focus on promotion of maternal health during the pre- and peri-conception period, pregnancy and postnatal period as well as the health of the offspring during the first two years of life. These interventions emerge from the national and WHO recommendations that take into account available evidence. In the health and nutrition interventions, attention has been given to the notable risk factors for poor birth and childhood outcomes, such as maternal anemia, undernutrition, maternal depression, hypothyroidism, diabetes, RTI and sub-optimal infant feeding and responsive child care practices.

This study aims to demonstrate that healthy growth and development can only be achieved when the environment is enabling and free from constraints. The findings will help understand the extent to which linear growth can be accelerated when all the nutritional and health care needs of the mother and the child are addressed. The study will also show the importance of providing interventions during the pre- and peri-conception period for improving linear growth. The design also enables us to examine the effect of maternal height reflecting intergenerational adversities, on the efficacy of interventions to improve birth and child outcomes. If successful, the study will reveal what is potentially achievable in terms of improvement in linear growth. The findings from this study will advance our scientific understanding and will be helpful in designing relevant programs in India and other similar low-middle-income settings.

There are strengths and unique features of this study. First, this study involves a factorial design with a large sample size which will help us clearly understand the importance of pre-and peri-conception interventions on key birth and growth outcomes with adequate power. Second, the women will be followed up from pre-conception, through pregnancy till childhood. This spectrum of follow up will help provide insights into the transition and inter connectivity of epidemiological, clinical, biochemical and biological data and their effect on important birth and child health related outcomes. Third, we would be able to understand the role of intergenerational effects in altering the effects of the interventions on growth and development of children. Fourth, majority of the interventions are delivered at home through trained study workers and the compliance to these interventions is observed when possible. Any queries are promptly addressed and problems resolved. The intention is to deliver the interventions with high quality so as to maximize the internal validity and generalizability of the study findings.

The study has some limitations. First, it is an individually randomized trial. A cluster randomized design may have been better suited considering that the majority of the interventions aim to influence the behavior and practices of the individuals. The design therefore, limits conduct of community mobilization activities which have been shown to be effective [75]. However, the individually randomized nature allows for factorial design which, in turn, provides an opportunity to examine the effect of pre- and peri-conception interventions on birth and growth outcomes. Second, it is difficult to implement the whole set of WASH interventions at the individual household level. Certain aspects such as improving sanitation facilities were not possible in an individually randomized design. Third, in spite of the body of evidence of the role of domestic violence among women, child neglect and child abuse in influencing birth and child growth and developmental outcomes, the interventions directly targeting these issues were not included in the package. The study investigators felt that intervening on such sensitive issues could have created resistance or other problems among the families and in the study communities.

## Conclusion

The findings emerging from the study will provide useful insights on the maximum achievable reductions in adverse birth outcomes and improvements in growth and development of young children from lower middle socio economic settings, when the majority of the known adversities are adequately addressed. The insights from the pregnancy and postnatal phase of the trial will help in the strengthening of the already existing maternal and child health programs in India and other low-middle-income settings whereas those emerging from the pre- and peri-conception period will provide reasonable grounds for policy discussions pertaining to development of pre- and peri-conception programs, which currently are largely non-existing.

## Trial status

**Fig. 3:**
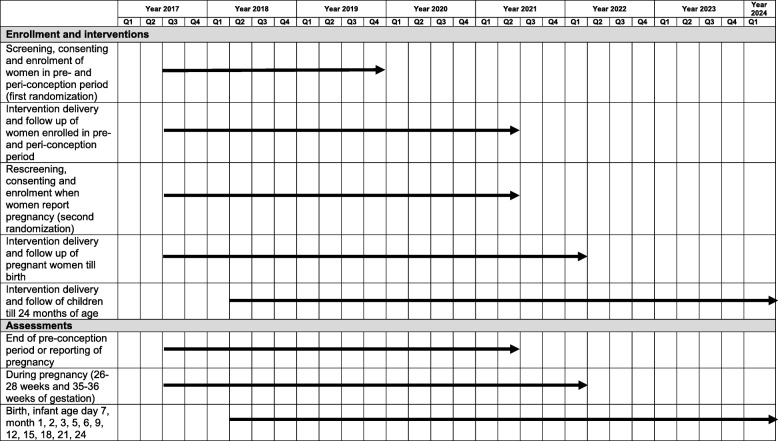
Schedule of Study Activities: SPIRIT Figure

The recruitment of participants commenced in July 2017 and enrollment will be completed by November 2019. Follow up visits will continue till enrolled women report pregnancy or complete 18 months in the pre- and peri-conception period. Pregnant women who provide consent are randomized again and followed up till their children are aged 2 years (Protocol version: 6.0 dated July 19, 2019; Fig. 3 and Additional file 4).

As the study duration is long, it is planned that learnings from the intervention group e.g. prevalence of non-communicable diseases, nutritional problems, infectious disease, different practices and behaviours in the study communities will be published to enable ongoing learning to be shared with policy makers.

## Supplementary information

**Additional file 1.** Technical Advisory Group.

**Additional file 2.** Details of interventions.

**Additional file 3.** Secondary outcomes in women and children and timing of measurement.

**Additional file 4.** SPIRIT 2013 Checklist: Recommended items to address in a clinical trial protocol and related documents.

## Data Availability

We are collaborators in the Healthy Birth, Growth, and Development Knowledge Integration (HBGDKi) initiative launched by the Bill & Melinda Gates Foundation. The data generated from the study will be shared as part of the HBGDKi repository (https://github.com/HBGDki) after study completion. The results of the research will be published in peer-reviewed biomedical journals. The findings will also be disseminated at conferences and communicated to the local and national health authorities, as well as to the World Health Organization.

## Abbreviations

AC: Abdominal circumference
AGP: Alpha-acid glycoprotein
ASHA: Accredited Social Health Activist
BIRAC: Biotechnology Industry Research Assistance Council
BMI: Body mass index
BPD: Biparietal diameter
CI: Confidence interval
CRL: Crown-rump length
DSMC: Data Safety Monitoring Committee
FL: Femur length
FTIR: Fourier transform infrared
GEE: Generalized Estimating Equation
GLM: Generalized linear model
Hb: Hemoglobin
HbA1c: Glycated hemoglobin
HC: Head circumference
IFA: Iron-folic acid
IUD: Intrauterine device
LAZ: Length-for-age z-score
LBW: Low birth weight
lOR: Interaction odds ratio
MUAC: Mid-upper arm circumference
OFD: Occipito-frontal diameter
PHQ-9: Patient Health Questionnaire-9
RDA: Recommended daily allowance
RPR: Rapid Plasma Reagin
RTI: Reproductive tract infections
SD: Standard deviation
SDGs: Sustainable Development Goals
SET: Screening and enrolment team
SGA: Small-for-gestation
STI: Sexually Transmitted Infections
TAG: Technical Advisory Group
TB: Tuberculosis
UNICEF: United Nations Children’s Fund
WASH: Water, Sanitation and Hygiene
WAZ: Weight-for-age z-score
WHO: World Health Organization
WINGS: Women and Infants Integrated Growth Study
WLZ: Weight-for-length z-score
