# Supplementary material for: Impact of an integrated nutrition, health, water sanitation and hygiene, psychosocial care and support intervention package delivered during the pre- and peri-conception period and/or during pregnancy and early childhood on linear growth of infants in the first two years of life, birth outcomes and nutritional status of mothers: study protocol of a factorial, individually randomized controlled trial in India
Source: Trials. 2020 Jan 31;21:127. doi: 10.1186/s13063-020-4059-z (PMC6995212; doi:10.1186/s13063-020-4059-z)
Supplement: Supplementary file 1 — Additional file 1. Technical Advisory Group. [file 13063_2020_4059_MOESM1_ESM.docx]

**ADDITIONAL FILE 1**

**Technical Advisory Group**

Dr. MK Bhan, National Science Professor, Indian Institute of Technology, New Delhi, India

Dr. HPS Sachdev, Consultant Pediatrician, Sitaram Bhartia Institute of Science and Research, New Delhi, India

Dr. Olufemi Oladapo, Medical Officer, Department of Reproductive Health and Research, World Health Organization, Geneva

Dr. Paul Hunter, Professor, Health Protection, University of East Anglia, United Kingdom

Dr. Reynaldo Martorell, Professor, International Nutrition, Emory University, USA

Dr. Margaret Montgomery, Technical Officer, World Health Organization, Geneva

### Dr. Vibha Krishnamurthy, Founder and Medical Director, [Ummeed - Child Development Center, Mumbai, India](http://ummeed.org/)

Dr. Mark Tomlinson, Professor, Department of Psychology, Stellenbosch University, South Africa

Dr. Rajiv Bahl, Department of Maternal, Newborn, Child and Adolescent Health, World Health Organization, Geneva

Dr. Nigel Rollins, Department of Maternal, Newborn, Child and Adolescent Health, World Health Organization, Geneva

Dr. Jose Martines, Independent Consultant, France

Dr. Shirshendu Mukherjee, Mission Director, Programme Management Unit, Biotechnology Industry Research Assistance Council (BIRAC), Department of Biotechnology, Government of India

Dr. Parul Christian, Senior Program Officer, Women’s Nutrition Global Health, Discovery & Translational Sciences, Bill & Melinda Gates Foundation, USA

Dr. Pratima Mittal, Professor and Consultant, Department of Obstetrics and Gynecology

Dr. Gautam Bhan, Lead (Academics & Research), Indian Institute of Human Settlements, Bangalore, India
