# Supplementary material for: Impact of an integrated nutrition, health, water sanitation and hygiene, psychosocial care and support intervention package delivered during the pre- and peri-conception period and/or during pregnancy and early childhood on linear growth of infants in the first two years of life, birth outcomes and nutritional status of mothers: study protocol of a factorial, individually randomized controlled trial in India
Source: Trials. 2020 Jan 31;21:127. doi: 10.1186/s13063-020-4059-z (PMC6995212; doi:10.1186/s13063-020-4059-z)
Supplement: Supplementary file 2 — Additional file 2. Details of interventions. [file 13063_2020_4059_MOESM2_ESM.docx]

**ADDITIONAL FILE 2. DETAILS OF INTERVENTIONS**

**Pre- and Peri-conception**

| **Intervention** | **Control** |
| --- | --- |
| **HEALTH**  Screen and treat medical conditions known to affect fetal and infant growth i.e. STI/RTI, TB, Hypertension, Diabetes and Pre-diabetes, Hypo and Hyper thyroidism  Treat those with medical conditions  Provision of contraception to women living with husband for <1 year, having child <1 year, severe malnutrition, severe to moderate anemia, hypothyroidism, RTI/STI and diabetes  Bi-annual deworming | Weekly IFA supplementation as part of the National Iron plus Initiative Program |
| **NUTRITION**  Weekly IFA supplementation (Iron 100 mg and folic acid 2400 mcg; Autrin®, Wyeth Pharmaceuticals Ltd), multiple micronutrients to all  All women screened for malnutrition and anemia  All women given multiple micronutrients (Vitamin A, C, B12, B6, B1, B2, Zinc, Selenium, Copper, Magnesium, Iodine- (Riconia Silver®, Sun Pharmaceutical Industries Ltd), IFA prophylaxis and nutritional counselling  **Malnutrition**  BMI <16 kg/m^2^: Refer to hospital and food supplementation and egg or milk (1000 kcal/day and 20-22 g protein/day)  BMI 16 to 18.40 kg/m^2^: food supplementation and egg or milk (500 kcal/day and 12-14 g protein/day)  BMI 18.5 to 20.99 kg/m^2^: Egg or milk (180 ml, 70kcal, 6 g protein)  **Anemia**  Severe anemia (Hb <8 g/dL): Hospital treatment  Mild to moderate anemia (Hb 8 to 11.99 g/dL): IFA treatment |  |
| **WASH**  Promotion of personal, menstrual and hand hygiene |  |
| **PSYCHOSOCIAL SUPPORT**  Screen all for depressive symptoms, substance abuse and exposure to second hand smoke  Promotion of positive thinking and problem-solving skills for all  Depressive symptoms (PHQ 9 ≥15 and /or presence of suicidal ideation): Refer to hospital |  |

Monitor all above interventions every 3 months until corrected or pregnant

Electronic monitoring system for tracking women with problems to support them for achieving intervention compliance across all domains.**Pregnancy**

| **Intervention Group** | **Control Group** |
| --- | --- |
| **HEALTH**  Minimum 8 ANC contacts for all according to WHO antenatal care guidelines  In addition to hospital based ANC clinics increase in coverage achieved by Free, High quality, Outpatient clinic within community with laboratory services, and use of electronic records to increase follow up  Screening and treat for medical conditions:  HIV, VDRL, syndromic STI/RTI, syndromic TB, HbsAg, hypo- and hyper-thyroidism at first contact  Urine R/E, M/E and asymptomatic bacteriuria by urine culture four times  Gestational diabetes by oral glucose tolerance test thrice  Pregnancy induced hypertension (blood pressure and urine protein at four times)  Anemia (Hb) four times  Increased quality of screening and treatment achieved by home based follow up, increased time per visit guided by individual electronic digital data record of continuity of follow up  Tetanus Toxoid immunization for all  Calcium and vitamin D supplementation daily starting from second trimester throughout pregnancy, for all  Anti-helminthic drug at 20 weeks | Routine antenatal care |
| **NUTRITION**  Counselling  IFA supplementation daily starting from second trimester throughout pregnancy for all  Multiple micronutrients (Riconia Silver®, Sun Pharmaceutical Industries Ltd) (Vitamin A, C, B12, B6, B1, B2, Zinc, Selenium, Copper, Magnesium, Iodine) daily throughout pregnancy  Daily locally-prepared snacks and support for women with BMI <25 kg/m^2^ at the time of second randomization  Second trimester: 280 kcal, 8 g protein (milk and snack)  Third trimester: 470 kcal, 27 g protein (milk and snack)  Weight monitoring at each month for all; management for inadequate weight gain (providing additional snacks; 500 kcal, 20 g protein).  Additional snack (500 kcal, 20 g protein) for pregnant women with BMI <18.5 kg/m^2^ at second randomization throughout pregnancy. |  |
| **WASH**  Provision of hardware (water filters and plastic bottles soap, hand washing station, disinfectant) and counselling |  |
| **PSYCHOSOCIAL SUPPORT**  Screen all for depressive symptoms, substance abuse and exposure to second hand smoke  Promotion of positive thinking and problem-solving skills for all; Thinking Healthy, World Health Organization  Depressive symptoms (PHQ 9 ≥15 and /or suicidal ideation): Refer to hospital |  |

Electronic monitoring system for tracking women with problems to support them for achieving intervention compliance across all domains.

**0-6 months (Infants)**

| **Intervention group** | **Control Group** |  |
| --- | --- | --- |
| **NUTRITION**  **For all infants**  Initiation of breastfeeding within the first hour of birth  Early lactation counselling for all mothers to prevent problems in BF in the first month after birth.  Breastfeeding problem resolution anytime during the first 6 months  Counsel on Exclusive breast feeding till 6 months of age and special  Emphasis on the exclusivity of breastfeeding from 3 up to 6 months of age.  **Growth monitoring and management of inadequate weight gain (IWG)**  Weight measurement at age day 14 and thereafter monthly to identify Inadequate Weight Gain (IWG) for all Term infants.  [IWG defined as <15^th^ centile as per WHO growth velocity, i.e. weight gain < 20 grams /day between ages Day 14 to 2 months; weight gain < 15 g/d for months 3 and 4; weigh gain <10 g/d for months 5 and 6)]  Intensified lactation support to all infants with IWG and thorough clinical examination to rule out possible causes for the same.  Facility based management of IWG by senior paediatrician at SJH after 15 days of continued efforts for lactation support and no medical cause is identified which may cause IWG.  Additional support for LBW babies and Preterm babies even if not LBW  Additional breastfeeding support by additional Lactation Counselling through home visits in the first three months (biweekly in first month, weekly in second and third month, monthly from fourth to sixth  month)  Offer expressed breastmilk feeding only for preterm babies after they breastfeed  Extended hospital support (through assessment of feeding, growth and investigations within 4-6 weeks after birth) after discharge and ensuring that the advice given at the facility is followed at home  Support kangaroo mother care at home  Vitamin D 400 IU daily for all infants up to 6 months  Iron supplementation at 2 weeks for VLBW and at 6 weeks for LBW | Routine postnatal and early childhood care |  |
| **HEALTH**  Educating the mother and other family members to identify danger signs and early care seeking for illness.  Facilitate referral to health facility for infants with any danger signs or illness requiring facility based management.  Counsel on timely immunisation |  | |
| **PSYCHOSOCIAL CARE**  Counselling, demonstration and practice sessions for mothers at each home visit on Early child play and responsive care.  Identify of delayed development and timely referral for further management |  |  |

Electronic monitoring system for tracking women with problems to support them for achieving intervention compliance across all domains.

**0-6 months (Mother)**

| **Intervention group** | **Control Group** |
| --- | --- |
| **HEALTH**  Facilitate mandatory Postnatal hospital visit at 6 weeks. | Routine postnatal and early childhood care |
| **NUTRITION**  **For all**  Daily locally-prepared snacks and milk, 600 kcal, 20g protein) for first 6 months of postnatal period.  IFA, Calcium and vitamin D supplementation daily for first 6 months of postnatal period  Multiple micronutrients (Riconia Silver®, Sun Pharmaceutical Industries Ltd) (Vitamin A, C, B12, B6, B1, B2, Zinc, Selenium, Copper, Magnesium, Iodine) daily for first 6 months of postnatal period |  |
| **WASH**  Continuation of all the WASH interventions as being provided during pregnancy (Water filters and plastic bottles soap, hand washing station, disinfectant) and counselling on handwashing and hygiene practices (bathing the infant regularly, using clean clothes for the infant, keeping the infant’s surroundings clean, safe disposal of infant’s feces, and handwashing before handling the baby). |  |
| **PSYCHOSOCIAL CARE**  Promotion of positive thinking and problem-solving skills for all; Thinking Healthy, World Health Organization  Screening for all mothers for depressive symptoms and management as required  Depressive symptoms (PHQ 9 ≥15 and /or suicidal ideation): Refer to hospital  Ascertainment of Substance abuse and exposure to second hand smoke and counsel against their use. |  |

Electronic monitoring system for tracking women with problems to support them for achieving intervention compliance across all domains.

**6-24 months (Children)**

| **Intervention group** | **Control** |
| --- | --- |
| **NUTRITION**  Effective counselling on initiation of complementary feeding by preparing the mother and family 1- 2 weeks prior 6 months of infants age  Initiation of Complementary feeding at 6 months of age and teaching the mother by demonstrating on how to prepare foods at home which can be fed easily to the child 6 months onwards.  Provide daily food supplement with 125 kcal/ 2.5 grams protein upto 12 months and 250 Kcal energy and 5 gram protein from 12 to 24 months and 80 to 100% RDA micronutrients throughout 6-24 months (Pristine 1^st^ Bite^®^, Pristine Organics Pvt Ltd, Bangalore, India)  Intake of home-based food  Counselling and demonstration of responsive feeding to mother and family members  IFA supplementation up to 2 years  Lactation counselling for supporting continued BF after 6 months  Growth monitoring (weight and length monthly); management of inadequate weight gain (<25^th^ centile as per WHO growth velocity) by providing additional supplements in form of snacks (~125 kcal and ~2.5 g protein upto 12 months and ~250 Kcal energy and ~5 g protein from 12 to 24 months)  Home based management of Moderate Acute Malnutrition (MAM) by providing counselling on preparing augmented home based foods.  Facilitating facility based management of Severe Acute Malnutrition (SAM). | Routine postnatal and early childhood care |
| **HEALTH**  Educate the mother and other family members to identify danger signs and early care seeking and facilitate medical management.  Counselling on feeding the child during and after illness.  Counsel on timely Immunization  Provide Albendazole (200 mg) for deworming starting 12 months of age and 6 monthly up to 24 months of age. |  |
| **WASH**  Continuation of all the WASH interventions as being provided during pregnancy (Water filters and plastic bottles soap, hand washing station, disinfectant) and counselling on hygiene practices (safe preparation, storage and feeding of the child utilising clean utensils and clean water for cooking and drinking).  Clean play area for children (play mat)  Safe disposal of child’s faeces (potty) |  |
| **PSYCHOSOCIAL CARE**  **CHILDREN**  Counselling on early child development  Demonstration and practice session for mother at each home visit on Early child play and responsive care.  Identify of delayed development and timely referral for further management  **MOTHERS**  Promotion of positive thinking and problem-solving skills for all; Thinking Healthy, World Health Organization  Screening for all mothers for depressive symptoms and management as required  Depressive symptoms (PHQ 9 ≥15 and /or suicidal ideation): Refer to hospital |  |

Electronic monitoring system for tracking women with problems to support them for achieving intervention compliance across all domains.
