# Supplementary material for: Impact of an integrated nutrition, health, water sanitation and hygiene, psychosocial care and support intervention package delivered during the pre- and peri-conception period and/or during pregnancy and early childhood on linear growth of infants in the first two years of life, birth outcomes and nutritional status of mothers: study protocol of a factorial, individually randomized controlled trial in India
Source: Trials. 2020 Jan 31;21:127. doi: 10.1186/s13063-020-4059-z (PMC6995212; doi:10.1186/s13063-020-4059-z)
Supplement: Supplementary file 3 — Additional file 3. Secondary outcomes in women and children and timing of measurement. [file 13063_2020_4059_MOESM3_ESM.docx]

**Additional File 3. Secondary outcomes in women and timing of measurement**

| **Outcomes** | **Time Points** | | | | | | |
| --- | --- | --- | --- | --- | --- | --- | --- |
|  | **End of pre- and peri-conception period or reporting of pregnancy** | **Gestational age 26-28 weeks** | **Gestational age 35-36 weeks** | **At birth or day 7** | **Month 2** | **Month 6** | **Month 12** |
| Birth interval | ✓ |  |  |  |  |  |  |
| BMI (weight; height measured at enrollment) | ✓ |  |  |  |  | ✓ | ✓ |
| Weight Gain |  | ✓ | ✓ |  |  |  |  |
| Symptoms of RTI/STI | ✓ |  | ✓ |  |  |  |  |
| Depressive symptoms | ✓ |  | ✓ |  | ✓ |  | ✓ |
| Inflammatory markers (C-reactive protein, Alpha-acid glycoprotein) | ✓ |  | ✓ |  |  | ✓ |  |
| Micronutrient status (vitamin A, D, B12, zinc, iron, folate and selenium) | ✓ |  | ✓ |  |  | ✓ |  |
| Anemia status | ✓ |  | ✓ |  |  | ✓ |  |
| Thyroid status | ✓ |  |  |  |  |  |  |
| Diabetes status | ✓ |  |  |  |  |  |  |
| Postpartum morbidity |  |  |  | ✓ |  |  |  |
| Pregnancy outcomes, Still birth |  |  |  | ✓ |  |  |  |

**Secondary outcomes in children and timing of measurement**

| **Outcomes** | | **Time points** | | | | | | | | | |  |
| --- | --- | --- | --- | --- | --- | --- | --- | --- | --- | --- | --- | --- |
|  | **At birth or day 7** | **Month 1** | **Month 3** | **Month 5** | **Month 6** | **Month 9** | **Month 12** | **Month 15** | **Month 18** | **Month 21** | **Month 24** | |
| Attained length |  |  |  |  | ✓ |  | ✓ |  |  |  |  | |
| Attained weight |  |  |  |  | ✓ |  | ✓ |  |  |  | ✓ | |
| Stunted |  |  |  |  | ✓ |  | ✓ |  |  |  |  | |
| Underweight |  |  |  |  | ✓ |  | ✓ |  |  |  | ✓ | |
| Wasted |  |  |  |  | ✓ |  | ✓ |  |  |  | ✓ | |
| Weight and length trajectories | ✓ | ✓ | ✓ | ✓ | ✓ | ✓ | ✓ | ✓ | ✓ | ✓ | ✓ | |
| Head circumference | ✓ |  |  |  |  |  | ✓ |  |  |  | ✓ | |
| Mid upper arm circumference |  |  |  |  | ✓ |  | ✓ |  |  |  | ✓ | |
| Body composition (sub sample) |  | ✓ |  |  |  |  |  |  |  |  |  | |
| Caregiver reported development outcomes (sub sample) |  |  |  |  |  |  | ✓ |  |  |  | ✓ | |
| Cognitive, language and motor scores (sub sample) |  |  |  |  |  |  |  |  |  |  | ✓ | |
| Mother-infant bonding (sub sample) |  |  |  |  | ✓ |  | ✓ |  | ✓ |  |  | |
| Micronutrient status (vitamin A, D, B12, zinc, iron, folate and selenium) |  |  |  |  |  |  |  |  |  |  | ✓ | |
| Anemia status |  |  |  |  |  |  |  |  |  |  | ✓ | |
| Inflammatory markers |  |  |  |  |  |  |  |  |  |  | ✓ | |
| Morbidity |  | ✓ | ✓ |  | ✓ |  | ✓ |  | ✓ |  | ✓ | |
| Hospitalizations |  | ✓ | ✓ |  | ✓ |  | ✓ |  | ✓ |  | ✓ | |
| Early initiation of breastfeeding | ✓ |  |  |  |  |  |  |  |  |  |  | |
| Exclusive breastfeeding |  | ✓ |  | ✓ |  |  |  |  |  |  |  | |
| Continued breastfeeding |  |  |  |  |  |  | ✓ |  | ✓ |  | ✓ | |
| Complementary feeding |  |  |  |  |  | ✓ | ✓ |  | ✓ |  | ✓ | |
